# Bulked segregant CGT‐Seq‐facilitated map‐based cloning of a powdery mildew resistance gene originating from wild emmer wheat (*Triticum dicoccoides*)

**DOI:** 10.1111/pbi.13609

**Published:** 2021-05-12

**Authors:** Qiuhong Wu, Fei Zhao, Yongxing Chen, Panpan Zhang, Huaizhi Zhang, Guanghao Guo, Jingzhong Xie, Lingli Dong, Ping Lu, Miaomiao Li, Shengwei Ma, Tzion Fahima, Eviatar Nevo, Hongjie Li, Yijing Zhang, Zhiyong Liu

**Affiliations:** ^1^ State Key Laboratory of Plant Cell and Chromosome Engineering Institute of Genetics and Developmental Biology The Innovative Academy of Seed Design Chinese Academy of Sciences Beijing China; ^2^ National Key Laboratory of Plant Molecular Genetics CAS Center for Excellence in Molecular Plant Sciences Shanghai Institute of Plant Physiology and Ecology Chinese Academy of Sciences Shanghai China; ^3^ University of Chinese Academy of Sciences Beijing China; ^4^ Institute of Evolution and the Department of Evolutionary and Environmental Biology University of Haifa Haifa Israel; ^5^ The National Engineering Laboratory of Crop Molecular Breeding Institute of Crop Sciences Chinese Academy of Agricultural Sciences Beijing China

**Keywords:** BSA, ChIP‐Seq, NLR, PAV

Powdery mildew, caused by *Blumeria graminis* f. sp. *tritici* (*Bgt*), is a widely occurring foliar disease of wheat worldwide. Wild emmer wheat (WEW, *Triticum dicoccoides*) (AABB, 2*n* = 4*x* = 28), the progenitor of the cultivated tetraploid and hexaploid wheat, is highly resistant to powdery mildew, and many resistance alleles were identified in this wild species. However, only *Pm41* that encodes a nucleotide‐binding leucine‐rich repeat (NLR) protein has been cloned using a map‐based cloning approach (Li *et al*., 2020). NLR proteins account for the major gene families of disease resistance genes (Kourelis and van der Hoorn, 2018) and are subjected to epigenetic regulation including histone acetylation and DNA methylation (Li *et al*., 2019). Only 31%–34% of wheat NLRs were shared across all genomes, and the numbers of unique NLR varied from 22 to 192 per wheat cultivar (Walkowiak *et al*., 2020). These presence–absence variations (PAVs) of NLRs are obstacles for isolating disease resistance genes based on reference genome sequences. Bulked segregant analysis (BSA), an approach for identifying genetic variations associated with disease resistance genes, has been proven effective in mapping and cloning wheat *Pm5e* gene in combination with next‐generation sequencing (NGS) technology (Xie *et al*., 2020). The combination of BSA and core genome targeted sequencing (CGT‐Seq), an approach to capture the major types of histone modification in the genome (Qi *et al*., 2018), might be used as an effective approach to capture sequence variations and PAVs in wheat NLRs between disease resistant and susceptible genotypes.

Powdery mildew resistance gene *MlWE18* was identified in common wheat line 3D249 (Figure 1a), an introgression derivative of WEW accession WE18 (G‐360‐M), and initially mapped within a 23.5 cM genetic interval on chromosome 7AL (Han *et al*., 2009). In order to fine mapping and cloning of *MlWE18*, a CGT‐Seq based BSA pipeline was developed (Figure 1b). We selected 30 homozygous resistant and 30 susceptible F_3_ families from the Xuezao × 3D249 mapping population to construct a pair of phenotypically contrasting DNA bulks. Chromatin immunoprecipitation sequencing (ChIP‐Seq) was performed for three histone marks H3K27me3, H3K4me3, and H3K36me3 that are closely associated with gene activity. After quality control, 423 924 608 and 358 879 346 reads for the resistant and susceptible DNA bulks, respectively, were obtained for subsequent analysis.

**Figure 1:**
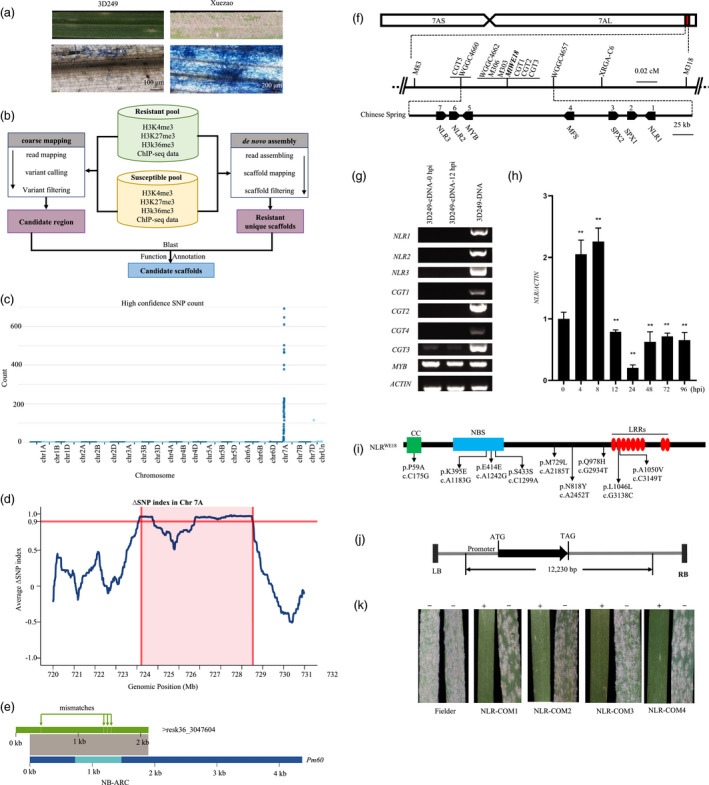
Map‐based cloning of powdery mildew resistance gene *MlWE18* introgressed from wild emmer into common wheat using a bulked segregant Core Genome Targeted sequencing (CGT‐Seq) approach. (a) Reactions and Coomassie Brilliant Blue staining of 3D249 and Xuezao to *Bgt* isolate E09. (b) Workflow of CGT‐seq based bulked segregant analysis. (c) The number of high‐quality SNPs that differ between ChIPed resistant and susceptible DNA pools. (d) △SNP‐index plot for the *MlWE18* genomic region. (e) The CGT‐Seq detected contig sequence (green) showed highly sequence similarity with *Pm60*. (f) High‐density genetic map of *MlWE18* and predicated genes in the 334 kb physical interval of Chinese Spring 7AL. (g) The RT‐PCR expression patterns of the predicted genes and the 4 CGT‐Contigs in the mapping interval in 3D249. (h) The relative expression levels of *NLR^WE18^* examined by qRT‐PCR at different h post inoculation (hpi) in 3D249. (i) Structure of the *NLR^WE18^* and sequence difference with *Pm60*. The positions of SNPs and amino acids variations in the CDS (c.) and NLR protein were illuminated, respectively. (j) Plasmid construct of *ProNLR^WE18^*:*NLR^WE18^* used for transformation. (k) Powdery mildew responses of *NLR^WE18^* transgenic T_1_ plants. +/−, presence/absence of the transgene.

Firstly, candidate chromosome region potentially associated with *MlWE18* was located. Following stringent mapping and filtering steps, 8,461 high confidence single nucleotide polymorphisms (SNPs) were detected (Figure 1c). The △SNP‐index method (Takagi *et al*., 2013) was applied to calculate the quantitative SNP differences between the two ChIPed DNA bulks for each of consecutive chromosome window. Further screening with regard to the read numbers and genotypes resulted in 5.32 Mb interval significantly associated with disease resistance on chromosome 7AL (724.21–729.53 Mb) (Figure 1d). To detect sequence variations associated with the resistance allele, the ChIPed sequences from the susceptible and resistant pools were de novo assembled, resulting in 655 192 and 685 858 contigs, respectively. The sequences specifically present in the resistant pool represented the candidate sequences within or near by the *MlWE18* locus. The resistant bulk‐specific contigs within the physical interval detected above were selected and mapped to the Chinese Spring (CS) reference genome. Since CS was susceptible to powdery mildew, we focussed on sequences that were partially mapped to CS (containing unmapped fragments, or with insertions or deletions). A total of 479 CGT‐Seq‐derived contigs were identified in the resistant pool and sequence annotation revealed that five contigs contained NB‐ARC domain, with lengths ranging from 595 to 2128 bp and sequence identities ranging from 15% to 56% between each other. Significantly, one 2128 bp contig (resk36_3047604) was highly homologous (99% identity) to the diploid *T*. *urartu* wheat *Pm60* sequence (Zou *et al*., 2018), with only 4 single nucleotide variations (SNVs) (Figure 1e).

In order to fine map the *MlWE18* locus, the five contigs identified by CGT‐Seq together with sequences from the 5.32 Mb 7AL physical intervals of CS were used to develop polymorphic markers (*CGT1*‐*CGT5*; *M83*‐*M405*) linked to *MlWE18*. Since *MlIW172* was mapped to the same chromosomal region as *MLWE18* (Ouyang *et al*., 2014), polymorphisms of markers *WGGC4660*, *WGGC4662*, and *WGGC4657* linked to *MlIW172* were also tested. After screening a mapping population of 1113 F_2_ plants (2226 gametes) derived from the Xuezao × 3D249 cross, three CGT‐Seq contig‐derived markers *CGT1*, *CGT2*, and *CGT3* were found to co‐segregated with *MlWE18*, whereas *CGT5* was closely linked (~0.05 cM) (Figure 1f). No polymorphism was detected between Xuezao and 3D249 for *CGT4*. *CGT1*, *CGT2*, and *CGT3* were dominant markers detected only in the resistant 3D249, but not in the susceptible Xuezao, CS, *T*. *urartu* G1812, and WEW Zavitan, indicating PAVs of these NLR‐like sequences between genotypes. Finally, *MlWE18* was mapped within a 0.09 cM genetic interval between markers *WGGC4660* and *WGGC4657*, and co‐segregated with *M306*, *M303*, *CGT1*, *CGT2*, and *CGT3* (Figure 1f), corresponding to a 334 kb genomic region in the CS 7AL reference genome. Annotation of the genomic region identified a MYB‐related protein, a major facilitator superfamily (MFS) protein, two Syg1/Pho81/XPRI (SPX) domain‐containing proteins, and three nucleotide‐binding sites with leucine‐rich repeats proteins named NLR1, NLR2, and NLR3 (Figure 1f). RT‐PCR was conducted to test the expression of the 7 predicted genes and the 4 CGT‐Seq derived contigs residing in the *MlWE18* genomic region. The results indicated that only the *TraesCS7A02G553800* (*MYB*) allele and *Pm60*‐related contig resk36_3047604 (CGT3, *NLR^WE18^*) were expressed in 3D249 before and after *Bgt* isolate E09 inoculation whereas the others were not (Figure 1g). Since contig resk36_3047604 was highly homologous to the *Pm60*‐encoded NBS‐LRR protein and was up‐regulated in 3D249 (Figure 1h), the *NLR^WE18^* allele in 3D249 was prioritized for validation as candidate for *MlWE18*.

The genomic DNA and full‐length cDNA sequences of *NLR^WE18^* were cloned from the resistant 3D249, and no alternative splicing variant was found in the gene body. The *NLR^WE18^* contained only one exon and encoded a typical CC‐NBS‐LRR protein with 1454 amino acids that was allelic to *Pm60* with six synonymous and three nonsynonymous SNPs (Figure 1i). To further validate the function of *NLR^WE18^* in resistance to *Bgt* isolate E09, construct *ProNLR^WE18^*: *NLR^WE18^* driven by the native promoter was transformed into the susceptible common wheat cultivar Fielder by *Agrobacterium*‐mediated (strain EHA105) transformation. The construct contained a 12 230 bp genomic fragment with 2103 bp of presumed native promoter, 4365 bp exon region, and 5762 bp terminator (Figure 1j). Four positive T_0_ transgenic plants with the confirmed transgene sequence were obtained. All of the positive transgenic T_0_ plants and their transgene‐positive T_1_ descendants were highly resistant to *Bgt* isolate E09 (Figure 1k). These results indicated that *NLR^WE18^* is functional powdery mildew resistance allele *MlWE18* (Genebank accession MW375697).

Most of the cloned wheat disease resistance genes are NLRs that tend to be PAVs between the resistance and susceptible genotypes, especially the genes derived from wild relatives (Li *et al*., 2020; Walkowiak *et al*., 2020). In this study, we cloned the powdery mildew resistance allele *MlWE18* (*NLR^WE18^*) whose locus is not present in the wheat reference genomes using a combined BSA and CGT‐seq strategies. The major advantage of this approach is that it is mostly reference genome free and can identify genes which are not present in the reference genomes but in a specific donor line. The proposed bulked segregant CGT‐Seq approach and the computational strategy can be used in further genetic mapping of genes controlling important agronomic traits in wheat and other crops with large genomes.

## Conflict of interest

The authors declare no conflict of interest.

## Author contributions

Z.L. and Y.Z. conceived the project. Q.W., Y.C., P.Z., H.Z., G.G., P.L., and M.L. performed fine mapping and map‐based cloning. F.Z. performed the CGT‐Seq experiment and data analysis. F.Z., J.X., L.D., and S.M. performed bioinformatic and micro‐collinearity analysis. T.F. and E.N. provided wild emmer wheat materials. Q.W., F.Z., H.L., T.F, Y.Z., and Z.L. wrote the manuscript.
